# Empagliflozin improves cardiac function in heart failure with reduced ejection fraction independent of loading conditions

**DOI:** 10.1186/s12933-020-01004-9

**Published:** 2020-03-10

**Authors:** Bo Liang, Yu-Xiu Zhao, Ning Gu

**Affiliations:** 1 Nanjing University of Chinese Medicine, Nanjing, China; 2 Hospital (T.C.M.) Affiliated to Southwest Medical University, Luzhou, China; 3 Nanjing Hospital of Chinese Medicine Affiliated to Nanjing University of Chinese Medicine, Nanjing, China

**Keywords:** Empagliflozin, Heart failure with reduced ejection fraction, Sodium-glucose linked cotransporter-2 inhibitor

## Abstract

The study regarding load-independent effects of empagliflozin contribute to improved cardiac function in experimental heart failure with reduced ejection fraction is very interesting. But there are a few things we need to pay attention to.

The present Commentary refers to the recently published article by Connelly et al. [1] describing that empagliflozin contributes to improving cardiac function in experimental heart failure with reduced ejection fraction (HFrEF). EMPA-REG OUTCOME demonstrated that patients with type 2 diabetes at high risk for cardiovascular events who received empagliflozin, as compared with placebo, had a lower rate of the primary composite cardiovascular outcome and of death from any cause when the study drug was added to standard care [2]. The underlying mechanisms are also being explored. It has been shown that empagliflozin improves hemodynamics in a hypertensive heart failure rat model, associated with renal protection, attenuated cardiac fibrosis, and normalization of HF genes [3]. Glycaemic control with empagliflozin significantly ameliorated myocardial oxidative stress injury and cardiac fibrosis in diabetic mice [4]. The drug also improves primary hemodynamic parameters and attenuates the progression of atherosclerosis by reducing hyperlipidemia and hyperglycemia [5] and reduces the levels of CD36 and cardiotoxic lipids while improving autophagy in the hearts of Zucker diabetic fatty rats [6]. Moreover, empagliflozin improves coronary microvascular function and contractile performance in prediabetic ob/ob^−/−^ mice [7] and attenuates ischemia and reperfusion injury through LKB1/AMPK signaling pathway [8]. We believe that it is important and necessary to analyze and report the potential underlying mechanism of empagliflozin for the benefit of heart failure, especially HFrEF, both load-dependent and load-independent effects. This study identified experimental HFrEF model through ligation of the left anterior descending coronary artery to induce myocardial infarction of the left ventricle to suggest that empagliflozin had major beneficial effects on the principal load-independent measures of systolic function, preload recruitable stroke work relationship and end systolic pressure volume relationship, indicating its salutary effects were, at least in part, due to actions beyond a direct effect of reduced preload and afterload [1]. But there are a few things we need to pay attention to. Firstly, this study established an experimental HFrEF model after myocardial infarction. Although this post myocardial infarction model develops structural hallmarks of HFrEF [9], there are many causes of HFrEF, a more ideal model of heart failure is warranted. In addition, following confirmation of infarct size with echocardiography 1-week post myocardial infarction, animals were then further randomized to receive the vehicle, or the sodium-glucose linked cotransporter-2 inhibitor, empagliflozin (20 mg/kg/day by gavage), for 6 weeks [1]. Prior to randomized administration, the authors applied echocardiography only to determine the infarct size but did not confirm the structure of the heart, ejection fraction value, and other typical features and phenotypes of HFrEF. Thirdly, 20 mg/kg/day of empagliflozin was administrated, this is really too much. Remember, the dose of empagliflozin is only 10 mg or 25 mg once daily in clinical trials [2, 10], translating less than 3 mg/kg/day for rats. Finally, this is an experimental study, that is to say, a report from nonhuman study. We need evidence from clinical trials to further confirm this.

## Data Availability

Not applicable.

## Abbreviation

HFrEF: Heart failure with reduced ejection fraction
